# Short- and Midterm Comparison of Platelet-Rich Plasma with Hyaluronic Acid versus Leucocyte and Platelet-Rich Plasma on Pain and Function to Treat Hip Osteoarthritis. A Retrospective Study

**DOI:** 10.3390/gels7040222

**Published:** 2021-11-19

**Authors:** Michelangelo Palco, Paolo Rizzo, Giorgio Carmelo Basile, Angelo Alito, Daniele Bruschetta, Maria Accorinti, Roberto Restuccia, Danilo Leonetti

**Affiliations:** 1 Department of Biomedical, Dental and Morphological and Functional Images, Section of Orthopedics and Traumatology, University of Messina, 98122 Messina, Italy; michelangelo.palco@gmail.com (M.P.); rizpaolo@gmail.com (P.R.); dleonetti@unime.it (D.L.); 2 Department of Biomedical, Dental and Morphological and Functional Images, University of Messina, 98122 Messina, Italy; danielebruschetta@gmail.com; 3 Complex Operating Unit of Physical and Rehabilitation Medicine and Sports Medicine, Policlinico Universitario G. Martino, 98124 Messina, Italy; alitomedical@gmail.com (A.A.); mariva81@hotmail.it (M.A.); roberto.restuccia@polime.it (R.R.)

**Keywords:** PRP, osteoarthritis, cartilage, degeneration, pain, gel, L-PRP, hyaluronic acid

## Abstract

Hip osteoarthritis (HOA) leads to pain and reduced function. The use of intra-articular injections based on corticosteroids, platelet-rich plasma (PRP), or hyaluronic acid (HA) is becoming a common symptomatic therapy for HOA. For the first time, we compare the effectiveness of plasma with a high concentration of platelets and leukocytes (L-PRP) with PRP+HA in patients with mild to moderate HOA. A total of 26 patients in each group were administered with either L-PRP or PRP+HA. Outcomes were evaluated at baseline, 3 months, and 1 year after the injection. The mean visual analog scale (VAS) and Harris hip score (HHS) within and between groups among different time points were compared using repeated measures ANCOVA (age set as a covariate). Both treatments were effective in reducing VAS, but not in significantly increasing HHS. In the group treated with L-PRP, VAS showed interaction between time and treatment (in favor of L-PRP). Pairwise comparison for treatment and time point evidenced a significant difference at 1-year follow-up between L-PRP and PRP-HA. Outcomes support the idea that both treatments may be effective in reducing pain, with maximal pain reduction achieved after 3 months. L-PRP showed better results in reducing VAS over time. Both treatments are effective at reducing pain in the short to medium term. L-PRP could be the treatment of choice due to a more marked effect over time. Nevertheless, further research is needed to better describe the clinical outcome of these formulations.

## 1. Introduction

Like other chronic joint diseases, hip osteoarthritis (HOA) leads to reduced quality of life and disability, mainly due to hip pain and functional limitation. The rapid increase in the prevalence of HOA makes it one of the most common joint diseases: the global prevalence of HOA is approximately 0.85%, higher in females (0.98%) than in males (0.70%), and consistently increasing with age [1]. Conservative approaches including lifestyle modification and weight loss, the use of orthoses, and footwear rehabilitation (with aerobic and muscle strengthening exercises) are considered effective and can be administered to reduce swelling, pain, and disability. Additionally, pharmacological therapy is considered an effective nonsurgical option for osteoarthritis management, with systemic nonsteroidal anti-inflammatory drugs (NSAIDs) or intra-articular injection of several formulations, discussed below [2]. Interestingly, compared with osteoarthritis of other joints, HOA has a distinct etiology, more markedly determined by chronic inflammation. Blocking proinflammatory pathways may reduce the degeneration of joint structures to achieve improved pain control and functionality. Intra-articular injections have the potential to deliver critical doses of therapy in the damaged area, lowering the incidence of systemic adverse effects and resulting in an immediate action [2]. Platelet-rich plasma (PRP) is a relatively new treatment that, in a similar way to hyaluronic acid (HA) and corticosteroids (CS), can be administered via intra-articular injection as a conservative treatment for osteoarthritis [2,3]. PRP is an autologous gel, based on a high concentration of platelets, which could potentially become of common use in clinical practice due to the ability of platelets to release anti-inflammatory biomolecules, which could promote soft tissue healing [4], for example, growth factors stored in platelet α-granules, playing a role in the regeneration of articular cartilage [5].

On the other hand, as concerns HA, the delivery of this biomolecule in the arthritic joint is called viscosupplementation, and is considered crucial to restore the mechanical properties of synovial fluid, exerting an analgesic, anti-inflammatory, and chondroprotective effect [6]. 

Pain control and improved joint function are the main aims of conservative therapy of osteoarthritis [7,8,9,10]. In recent years, several formulations of PRP have been made available to manage osteoarthritis; nevertheless, adequate clinical evidence supporting the choice of a specific product over another is still lacking [3]. In vitro, leukocyte-poor PRP with leukocyte and platelet-rich plasma (L-PRP) attenuates the production of inflammatory cytokines; nevertheless, it reduces the chondrocytic production of HA. Therefore, it has been suggested to combine an anti-inflammatory molecule PRP with heterologous HA to guarantee viscosupplementation and contrast the depletion of autologous HA production [3,11,12]. The effectiveness and preferability of this new product are still controversial [13].

As regards HOA, this is the first study comparing clinical outcomes of PRP plus hyaluronic acid (PRP+HA)—which is relatively poor in leucocytes—with L-PRP, which conversely shows abundance of white cells. The primary aim of this study is to evaluate both L-PRP and PRP+HA treatments’ effectiveness to reduce pain and increase hip function. The secondary aim is to find out whether one of the two treatments could lead to better results: we hypothesized that, due to the viscosupplementation effect of HA, PRP+HA may show superior results in pain control in HOA with chronic pain.

## 2. Results and Discussion

### 2.1. Descriptive Statistics

Among 89 patients treated with L-PRP or PRP+HA, 27 did not meet the inclusion criteria: 26 patients were excluded due to a previously mentioned clinical condition. A patient treated with PRP+HA underwent total hip surgery. No patient refused informed consent.

**Figure 1:**
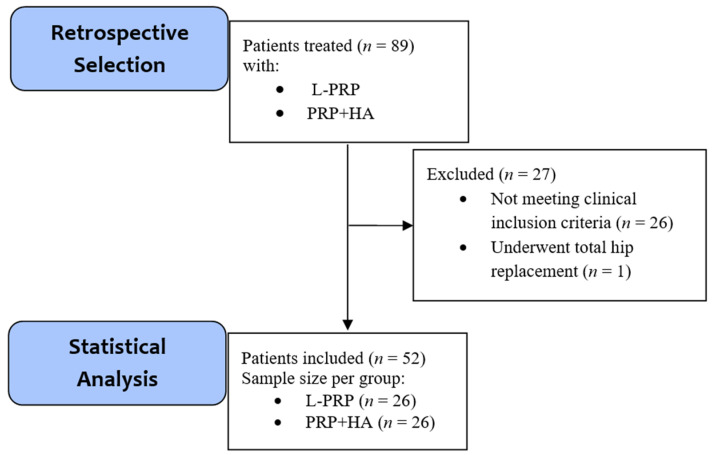
Disposition diagram summarizing the retrospective selection of patients, the number of patients excluded, and the final sample size obtained. *n* = number of subjects. L-PRP = leukocyte and platelet-rich plasma, PRP+HA = platelet-rich plasma + hyaluronic acid.

We included 52 patients suffering from HOA treated with intra-articular injections of L-PRP or PRP+HA between December 2017 and December 2018, meeting the inclusion criteria. Among these, 26 were treated with L-PRP and 26 with PRP+HA; from this point, we will address two separate groups (L-PRP and PRP+HA). Participants included in the analysis received the treatment, and none of them noticed/complained of any harmful/unexpected effects. A disposition diagram summarizes patient selection (Figure 1).

At the baseline moment, 24 patients (46.2%) showed II KL grade, while 28 patients (56.8%) showed III grade. The study included 52 patients for the final analysis, 24 females (46.2%) and 28 males (56.8%). Study participants treated with PRP+HA showed higher age (*p* = 0.001). Table 1 is a summary of the baseline characteristics of the two groups (Table 1).

### 2.2. Efficacy of L-PRP and PRP+HA

Clinical outcomes are summarized in Table 2.

No adverse effects were observed, apart from sporadic transient burning/stinging/warmth or swelling sensation in the injection site, which usually spontaneously resolved on its own within 15–60 min.

ANCOVA for VAS showed significant results (F (2, 98) = 13.836, *p* < 0.001, η_p_^2^ = 0.220), but not for HHS (*p* > 0.05). Post hoc Bonferroni pairwise comparison highlighted significant improvement in VAS between baseline and 3 months’ follow-up, the improvement in both measures was not retained, and between 3 months’ follow-up and 1-year follow-up, a significant worsening (increased VAS and reduced HHS) was observed (*p* < 0.001).

### 2.3. Differences between Treatments

In the L-PRP group, VAS showed significant changes (F (2, 98) = 3.286, *p* = 0.040, η_p_^2^ = 0.120). In PRP+HA, (F (2, 98) = 10.732, *p* < 0.001, η_p_^2^ = 0.309).

In the VAS score, no main effect of treatment type was highlighted (*p* > 0.001). As regards VAS scores, the interaction between time and treatment was significant (in favor of L-PRP), with a small effect size (F (2, 98) = 5.392, *p* = 0.007, η_p_^2^ = 0.099). Pairwise comparison for treatment and time point evidenced a significant difference at 1-year follow-up between L-PRP and PRP+HA (*p* = 0.020).

This work retrospectively compares pain and functional outcomes of patients suffering from mild/moderate HOA (KL stages II–III) undergoing two consecutive L-PRP or PRP+HA intra-articular injections 3 months and 1 year after treatment. 

A significant short-term reduction of pain of the osteoarthritic hip, as shown by the significant lowering of VAS, was the most important consequence of both treatments: on average, in the L-PRP group, the mean VAS decreased to 42.79 (baseline = 72.79; 3 months’ follow-up = 30.00), while in the PRP+HA group, it decreased to 32.30 (baseline = 70.38; 3 months’ follow-up = 38.08). Nevertheless, at the 1-year follow-up, the mean VAS worsened (L-PRP: +19.62; PRP+HA: +21.15): it was worsening when compared with the score after 3 months, but at the same time, it was significantly lower than baseline; in other words, the analgesic effect partly persisted after 12 months, but it was weaker than in the first follow-up visit. The group treated with L-PRP showed a lower mean VAS at the last follow-up (−9.61): results of our statistical analysis confirmed that there was a significant interaction between time and treatment, with significantly lower scores in the L-PRP group at the last follow-up. This observation suggests that L-PRP could induce a more marked long-term pain reduction compared with PRP+HA; nevertheless, it is important to consider the small effect size of this result as a limit of this observation, especially considering the restricted sample size of this study.

**Figure 2:**
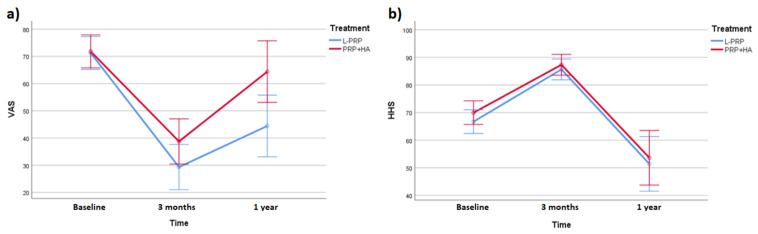
Profile plot of VAS and HHS at the different time points of the study in the two groups. Data are presented as estimated marginal means with 95% confidence interval. (**a**) shows a marked difference between average VAS score between the two groups. Difference is less evident (**b**). VAS = visual analog scale, HHS = Harris hip score.

Our results do not support the effectiveness of L-PRP and PRP+HA to improve function (walking and activities of daily living), reduce movement restrictions (limitations in hip flexion, adduction, and internal rotation), and improve ROM in flexion, abduction, adduction, and external rotation, measured using an orthopedic goniometer. It is worthy to note that, although the change in HHS in the two groups was found to be not significant, the evolution of this functional score in the two groups followed the same pattern of VAS: an initial (significant) improvement after 3 months, followed by a marked worsening between 3 months and 1 year (Figure 2). Despite a functional score change not being significant in our study, a recent meta-analysis reported a significant improvement in several patient-reported outcomes up to 1 year, with peak effectiveness between 4 and 6 months, with a similar pattern with the one shown in our study. The use of intra-articular therapy is supported by several lines of research in the field [14]: nevertheless, the picture becomes less clear if we take into account that, beyond PRP, HA, and CS, even intra-articular saline solution showed comparable effectiveness up to 6 months, shedding light on a possible placebo effect linked to intra-articular therapy, and advocating against a possible regenerative effect of these therapies [15]. This is important, especially if we take into consideration that saline solution is commonly labeled as “placebo” in studies taking into consideration intra-articular treatments. The evidence that PRP, CS, and HA has a placebo effect should not be considered conclusive, as it must be considered that the equivalence of saline solution and these therapies has been studied in only 11 studies, taking into consideration 6 months of follow-up. Furthermore, there is no agreement on whether it can be accurate to consider intra-articular saline solution as a placebo: it determines a substantial pain reduction, especially in a short time span, as shown by several studies primarily on patients with knee osteoarthritis [16,17].

Further evidence supporting the short-term efficacy of biological gel injections as a strong analgesic solution for HOA comes from a previous paper by Sante and colleagues: in a comparative study, either PRP or HA intra-articular injections were administered to a total of 43 patients suffering from severe monolateral HOA. PRP determined a 4-week pain reduction (assessed with VAS), which was not retained at the successive follow-up; conversely, the administration of HA determined a 16-week pain reduction [18]. Another work by Dallari and colleagues described the effects of PRP, HA only, and PRP+HA on patients with all grades of the K-L scale. The treatments were successful in reducing pain and improving function (assessed by WOMAC) in the first 6 months; after, only the analgesic effect lasted up to the 12 months’ follow-up. PRP was the formulation with the strongest pain-relieving effect. VAS scores were lowest at 6 months’ follow-up [19]. 

Currently, scientific evidence is supporting the view that the administration of intra-articular PRP injections could exert an analgesic effect more markedly within 3 months, with several reports of effectiveness in short-/midterm evaluations. The superiority of PRP against other injective treatments is yet to be determined, while longer-term evaluations from 4 to 12 months show diverse results [3]; despite this, it is reasonable to consider PRP a useful analgesic in light of the current data [3].

Our work, with a major focus on the comparison between L-PRP and PRP+HA, shows substantial agreement with the evidence brought on by previous research. Furthermore, in our study no relevant adverse effects were observed, supporting the idea that intra-articular injections are a safe conservative local analgesic treatment when administered by physicians with expertise in eco-guided intra-articular injections. PRP is widely considered beneficial and safe for patients with HOA, and PRP has been demonstrated to improve HOA symptoms at midterm follow-up [19,20]; nevertheless, high-quality studies are still necessary to assess whether it will become a routine part of the management of patients with HOA. PRP is, *de facto*, a relatively cheap solution, and it is not burdened by the collateral effects of systemic treatments. It can be obtained with ease and in a restricted amount of time and can be eventually delivered in the synovial cavity a few minutes after blood sampling [3,19,20]. In our opinion, it may become a conservative treatment of choice in the management of osteoarthritis in the next few years.

The interpretation of the results must take in consideration several limitations: due to the retrospective design, the baseline scores are obtained from a singular assessment of a nonrandomized group. Herein, design and statistical analysis are not accounting for regression to the means. We can only obtain preliminary conclusions, while a randomized study carried out with a test–retest design would be more indicated to compare the two treatments. Furthermore, we enrolled participants not responding to analgesic/anti-inflammatory use. Nevertheless, we did not systematically collect concomitant treatment, and we interviewed patients about their drug consumption (to exclude routine analgesic/anti-inflammatory drugs users): we did not take into account the occasional assumption of analgesic/anti-inflammatory drugs (e.g., NSAIDs).

The absence of follow-up after 1 year is a limitation to this study, as at that time point, the pain reduction was observable, but we did not assess whether this effect lasted more with one of the two treatments.

Being that this is a retrospective study, we did not compare the effects with other intra-articular treatments (e.g., PRP only or L-PRP+H) or with saline solutions. PRP preparations could vary from different manufacturers, and our results might not be extrapolated to all commercially available PRP preparations, but only those that determine the production of a PRP of similar composition.

We partially accounted for selection bias by adjusting for age; nevertheless, an analysis based on matching, if feasible (e.g., prospective setting), may have been superior.

## 3. Conclusions

To summarize, what we exposed previously, the two formulations analyzed, showed a significant analgesic effect: 1 year after the infiltrative therapy, pain perception was still improved, but the analgesic effect reached a peak in the baseline at 3 months’ interval.

In our opinion, these results encourage the adoption of PRP intra-articular administration as a safe, simple, and minimally invasive solution to mid- and long-term chronic pain management.

The efficacy of these and similar biological treatments and the comparison (in terms of effectiveness) with other nonsurgical options, as well as their possible role in severe OA or in other degenerative joint diseases management, could be better described by further studies.

Our result suggest that L-PRP may in fact determine, at a long-term follow-up, a superior pain reduction. Indeed, these results may not be enough to indicate a management choice, and more evidence is necessary to find out whether one, between L-PRP and PRP+HA, could be more indicated.

## 4. Materials and Methods

### 4.1. Study Design

#### 4.1.1. Patient Selection

This retrospective, comparative, observational study was conducted at the Policlinic “G. Martino” of Messina (Italy) to investigate outcome differences between PRP+HA and L-PRP. The study is under the authorization of the Comitato Etico di Messina, the local committee (registration number: 0014580). 

Participants were selected among patients suffering from HOA, treated with L-PRP or PRP+HA injections between December and November 2017 and December 2018. Standard follow-up for patients lasted 12 months, so data collection for the last patient enrolled in the study ended in December 2019.

Diagnosis was based on clinical history, physical examination, and weight-bearing anteroposterior radiographs of both knees in full extension, graded by adopting the Kellgren and Lawrence (KL) grading system [21] by two extensively trained physicians/researchers with several years of experience in the orthopedic field (M.P., D.L.). According to KL, HOA can be considered doubtful (grade I), mild (grade II), moderate (grade III), or severe (grade IV). 

Injective treatment was indicated in patients suffering from HOA, with physiological hematocrit and coagulation profile (between 150,000 and 450,000 platelets/mm^3^). Patients were considered eligible if they reported no pain relief after the repetitive administration of NSAIDs. Therefore, patients enrolled in this study were informed to avoid cortisone/NSAIDs for analgesic–anti-inflammatory purposes.

Patients with hematological/oncological diseases or with any of the following condition—history of spine or lower limb surgery, recent hip injuries, joint infections, bone necrosis, use of corticosteroids in the last 3 months before evaluation, routine use (e.g., due to comorbidities) of drugs, or physical therapy (e.g., cryotherapy) for analgesic/anti-inflammatory purposes—were excluded due to their influence on pain and function.

We included in our revision patients with a mild–moderate grade of HOA.

All patients received an ultrasonography-guided injection in the most symptomatic hip, followed by another one after 14 days for a total of two injections (one on the 1st day and another on the 15th day). Physiotherapy and gradual muscle strengthening have been prescribed since the second infiltration of the treatment.

Due to the retrospective nature of the study, patients were aware of the treatment received; thus, this can be considered an open-label work. Questionnaires were administered and collected by physicians external to the context of the study. Patients were asked for permission to use data collected in the study time after questionnaire administration, so the filling of questionnaires was not performed under research—but only clinical regime.

Statistical analysis was performed without any possible identifier of the groups (only named groups A and B).

#### 4.1.2. Injection Procedure

With the patient in supine position (lower limbs in neutral rotation), the physician sits on the side of the patient’s symptomatic hip.

The first step of the procedure is a survey sonography of the hip joint (ultrasound: MyLab™X7 system, Esaote S.P.A., 2021, with a convex transducer, Ac2541, 1–8 MHz frequency) to identify the anterior rim of the acetabulum, femoral head, femoral neck, and joint capsule, with Doppler imaging to identify femoral vessels.

The injection must be performed using aseptic technique. The patient’s groin area and anterior hip are sterilized with a chlorhexidine swab, and sterile ultrasound gel is applied over the injection site.

After cleaning the transducer using a germicidal cloth wipe, the hip joint is visualized in a long-axis view. Then a 3.5-inch 22-gauge sterile spinal needle is inserted 1 cm from the distal portion of the ultrasound transducer. The needle can be inserted, under visualization, in plane with the transducer until it enters the joint capsule.

The needle enters the hip joint near the anterolateral surface of the femoral neck, at the femoral head–neck junction. Injection is performed as lateral as possible to avoid lesion of femoral vessels.

**Figure 3:**
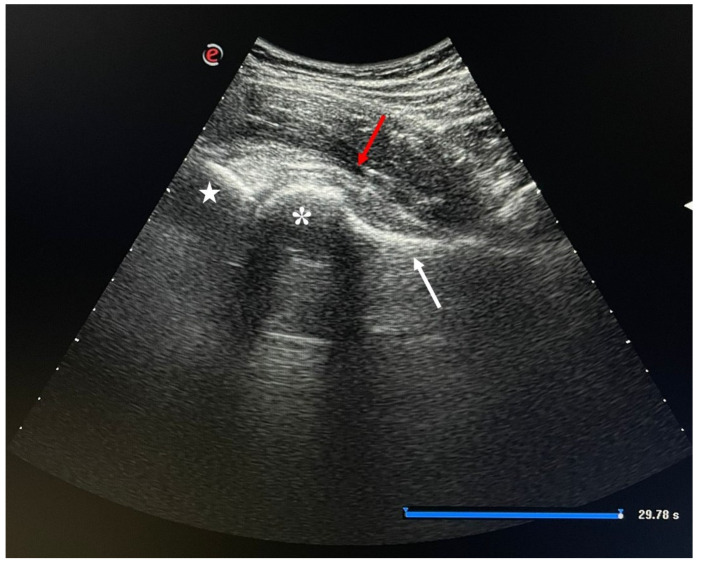
Ultrasound long-axis view of the hip as it is visualized during injection. Star = anterior rim of acetabulum; * = femoral head; white arrow = femoral neck; red arrow = joint capsule.

Holding the needle in the correct position, L-PRP or PRP+HA can eventually be injected (Figure 3). 

#### 4.1.3. Treatment

For each PRP injection, 8 mL of venous blood from the cubital vein was collected in a sterile tube. Subsequently, with RegenKit^®^-THT-3/RegenCell^®^ (Regen Lab SA, En Budron B2, 1052 Mont-sur-Lausanne, Switzerland) we obtained L-PRP, after centrifugation of venous blood for 9 min at 3400 rpm/1500× *g*.

Using Cellular Matrix A-CP-HA, we obtained PRP+HA with centrifugation (5 min; 3400 rpm/1500× *g*). The volume obtained from each tube stood at 5 mL (in the case of PRP+HA, 5 mL equals 3 mL of PRP and 2 mL of HA).

Performance tests were carried out by Regen Lab SA (following the U.S. Food and Drugs Administration requirements for PRP medical devices). Cellular Matrix A-CP-HA had a platelet concentration factor of 1.6×, with a platelet recovery of approximately 80%. PRP+HA reached a mean concentration of 310.000 platelets/mm^3^ and 1000 leukocytes/mm^3^ (in the PRP fraction). The Regen THT concentration factor was 1.7×. Platelet recovery was approximately 95%. L-PRP had a mean concentration of 370,000 platelets/mm^3^ and 4000 leukocytes/mm^3^.

Functionality tests performed in our hospital (with the contribution of 10 healthy volunteers, between 25 and 56 years of age) revealed that PRP+HA contains, on average, 800 leukocytes/mm^3^ and 290.000 platelets/mm^3^, while L-PRP contains 3600 leukocytes/mm^3^ and 340.000 platelets/mm^3^. The mean concentrations were calculated by an automatic cell counter (BC 6800, Auto Hematology Analyzer, Mindray, Shenzhen, China).

#### 4.1.4. Outcomes

**Figure 4:**
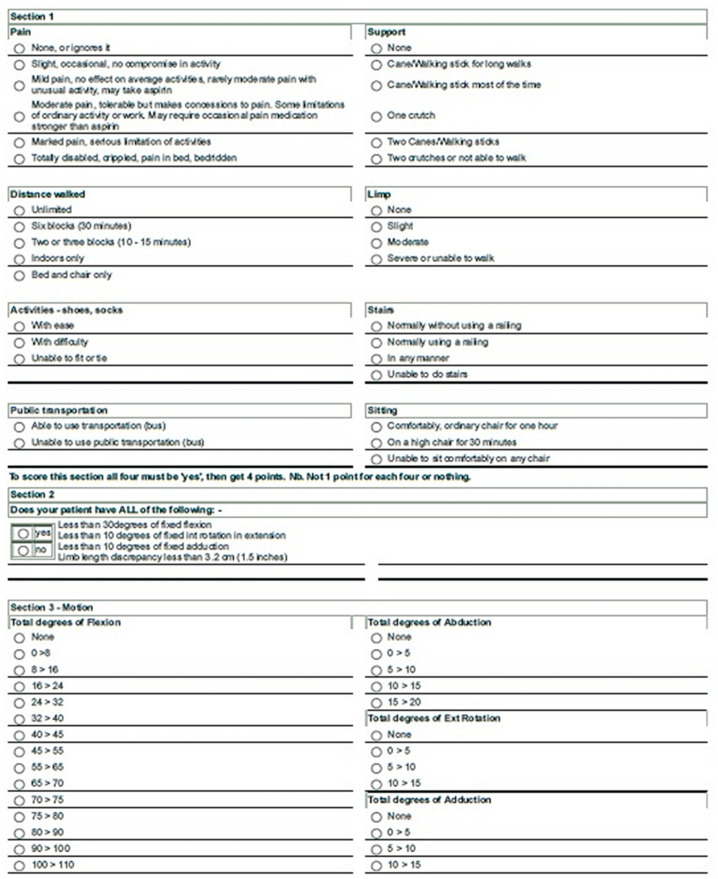
The scoring table for HHS, as it was administered in this study. Section 1 calculates a score based on pain and several functions, determined by lower-limb functionality, as reported by the patient. Section 2 is based on physician evaluation of hip range of movement and limb discrepancy. Section 3 is based on an accurate evaluation of any hip movement in the three dimensions.

To assess the effectiveness of the treatment on hip function and pain, we evaluated the patients with the Harris hip score (HHS) and the visual analog scale (VAS). The HHS consists of four subscales: pain severity (44 points); function, evaluating daily activities and gait (47 points); deformity, which measures hip flexion, adduction, internal rotation, leg length discrepancy, and range of motion measures (4 points); and range of motion (5 points). Scores range from 0 to 100. A score of <70 is considered a poor result, 70–80 is fair, 80–90 is good, and a score of 90–100 is excellent [22,23]. The reliability of the Italian version was determined by the ICC (interclass correlation coefficients), which showed high results for both interobserver reliability and test–retest with values of 0.996 and 0.975, respectively. The internal consistency measured with Cronbach’s alpha reached a value of 0.816, perfectly within the required range (0.7–0.95) [24]. The HHS was administered as shown in the following figure (Figure 4). 

We proposed to the patients the visual analog scale (VAS) on a segment of 100 mm, with “no pain” and “worst pain imaginable” as anchor descriptions. The patient was invited to place a sign to indicate the level of pain perception: the outcome is represented by the distance between the left (no pain) endpoint and the sign, measured in mm.

Participants were evaluated with VAS and HHS before injection (baseline evaluation, T0) and 3 months (T1) and 12 months (T2) after intra-articular injections. Treatment and outcome assessment were performed in outpatient regime by an orthopedic surgeon not involved in other procedures (PR).

#### 4.1.5. Statistical Analysis 

Sample size was assessed by G*Power software (University of Düsseldorf, Düsseldorf, Germany). A minimal sample size of 44 subjects (22 per group) was adopted, with α level = 0.05, power (1-β) = 0.95, and effect size of 0.25 (medium effect size) [25,26].

The differences between the two groups for gender, K-L grade (χ2 test), and mean age (independent-sample *t*-test) were analyzed. The two groups were compared for baseline HHS and VAS using independent-samples *t*-test. A general linear model (repeated measures ANCOVA) was adopted, based on the mean values of the outcome variables (HHS, VAS) between the levels of a within-subject and a between-subject factor (within: time; between: treatment), with simple main effects and interaction assessment. Post hoc Bonferroni pairwise comparison was performed. Age was set as a covariate. 

## Figures and Tables

**Table 1:** Demographic data of the patients in the study. L-PRP = leukocyte and platelet-rich plasma, PRP+HA = platelet-rich plasma + hyaluronic acid. Values in bold showed significant difference.

Treatment Group		L-PRP	PRP+HA	Total
**Age**		**50.62 ± 16.14**	**64.81 ± 10.81**	**57.47 ± 15.20**
Sex	M/F	16/10	12/14	28/24
KL grade	II/III	14/12	10/16	24/28

**Table 2:** Outcomes are reported with mean ± standard deviation (M ± SD). Results showing significant changes (by repeated measures ANCOVA, adjusted for age) are reported in bold characters (*p* < 0.05). FU = follow-up.

	Baseline	3 Months FU	1 Year FU
**VAS (L-PRP)**	**72.69 ± 13.65**	**30.00 ± 16.37**	**49.62 ± 14.53**
**VAS (PRP+HA)**	**70.38 ± 15.55**	**38.08 ± 19.90**	**59.23 ± 26.74**
HHS (L-PRP)	66.86 ± 9.96	86.09 ± 8.88	53.08 ± 24.31
HHS (PRP+HA)	69.93 ± 10.31	86.88 ± 8.88	51.97 ± 22.91

## Data Availability

Data are available on request.
